# Adversity magnifies the importance of social information in decision-making

**DOI:** 10.1098/rsif.2017.0748

**Published:** 2017-11-29

**Authors:** Alfonso Pérez-Escudero, Gonzalo G. de Polavieja

**Affiliations:** 1 Physics of Living Systems, Department of Physics, Massachusetts Institute of Technology, Cambridge, MA, USA; 2 Cajal Institute, Consejo Superior de Investigaciones Científicas, Madrid, Spain; 3 LAPLACE, Université Paul Sabatier, Toulouse, France; 4 Champalimaud Neuroscience Programme, Champalimaud Center for the Unknown, Lisbon, Portugal

**Keywords:** decision-making, collective behaviour, collective decisions, panic

## Abstract

Decision-making theories explain animal behaviour, including human behaviour, as a response to estimations about the environment. In the case of collective behaviour, they have given quantitative predictions of how animals follow the majority option. However, they have so far failed to explain that in some species and contexts social cohesion increases when conditions become more adverse (i.e. individuals choose the majority option with higher probability when the estimated quality of all available options decreases). We have found that this failure is due to modelling simplifications that aided analysis, like low levels of stochasticity or the assumption that only one choice is the correct one. We provide a more general but simple geometric framework to describe optimal or suboptimal decisions in collectives that gives insight into three different mechanisms behind this effect. The three mechanisms have in common that the private information acts as a gain factor to social information: a decrease in the privately estimated quality of all available options increases the impact of social information, even when social information itself remains unchanged. This increase in the importance of social information makes it more likely that agents will follow the majority option. We show that these results quantitatively explain collective behaviour in fish and experiments of social influence in humans.

## 1. Introduction

In many social species, individuals in a group make choices that are more similar to each other when conditions are adverse than when they are favourable. This phenomenon is well documented in groups of moving animals, which often form tighter groups in response to the detection of a predator [1–6]; but it also takes place in other adverse conditions, such as in the absence of food [7] or when animals are introduced into an unknown environment [8,9]. In humans, the occurrence of sudden bank runs [10–12] and human stampedes [13–19] suggests increased aggregation in adversity, although data are insufficient to draw definitive conclusions. We will use the term *super-aggregation in adversity* to describe all these situations, regardless of whether aggregation occurs in physical space (as in groups of prey fleeing from a predator) or in the space of possible choices (as in investors deciding what shares to buy or sell).

**Figure 1.**
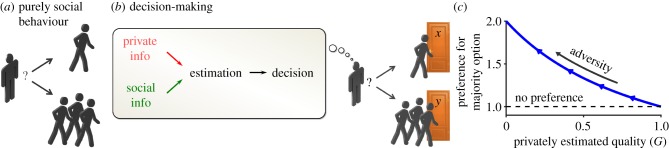
Super-aggregation in adversity emerges from estimation-based decisions. (*a*) Scheme of a purely social decision. The focal individual must decide which group to join and only takes into account the groups themselves. (*b*) Scheme of a more complete decision-making process. The deciding individual must choose which group to join, but these groups are also associated with their non-social circumstances (in this example, they are choosing different doors). (*c*) Bayesian decision-making predicts super-aggregation in adversity: preference for the majority option (defined as *P*(*Y* | *B*, *C*)/*P*(*X* | *B*, *C*) in equation (2.4)), as a function of the privately estimated quality for both options (*G* = *P*(*X* | *C*) = *P*(*Y* | *C*)).

Many theories study this phenomenon from a purely social point of view, investigating why an individual benefits from the company of its conspecifics—the intrinsic benefits of belonging to a group (figure 1*a*). For example, both theory and experiments show that belonging to a group may protect against predators [6,20–23]. In humans, copying others' behaviour can help to deflect the responsibility for an anticipated failure [24,25]. These explanations focus on cases in which aggregation holds an intrinsic benefit (such as collective defence from a predator), and require that this benefit increases in adversity—an increase that is often not obvious. Here we ask whether super-aggregation in adversity may emerge even in the absence of this benefit.

An indirect advantage of being in a group is that the behaviours of others can inform about the environment. Therefore, even if belonging to a group holds no intrinsic value, copying others' decisions may be the optimal strategy when reacting to an uncertain environment, and this strategy may lead to aggregation. The optimal level of aggregation resulting from this strategy can be calculated using decision-making theory (figure 1*b*). These types of models successfully explain collective behaviour in many animals, including humans [26–31], but have never been used to investigate super-aggregation in adversity.

Here we show that Bayesian estimation predicts super-aggregation in adversity, which was inadvertently present in some decision-making models [30,31], and absent from others due to simplifying assumptions [26–29]. Furthermore, we find that many non-Bayesian decision-making models may also lead to super-aggregation in adversity, and discuss the conditions in which this will hold. Using simulations, we show that the effect can be strong enough to reproduce typical observations of groups of animals under predation risk, and that its predictions are consistent with existing experimental data.

## 2. Results

Consider the example of a person who wants to exit a building, and must choose between two identical looking doors (*x* and *y*; figure 1*b*). We define the quality of each option as the probability that it leads safely to the exit. Even assuming that the presence of other individuals holds no intrinsic value, their behaviour may give information about the quality of the two doors: assuming that part of the people also want to exit the building, and that they may have some information about the correct path, a door chosen by the majority has a higher probability of being the correct option.

Bayes' theorem allows computing this probability combining social and private information. According to this theorem, the probability that door *y* is a good option is2.1
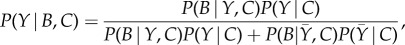
where *Y* stands for ‘*y* is a good option’ and 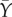 stands for ‘*y* is a bad option’. *B* and *C* represent the social and private information, respectively. *P*(*Y* | *C*) is the probability that option *y* is good, estimated using only private information; we therefore define the non-social term as 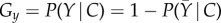. The terms *P*(*B* | *Y*, *C*) and 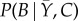 contain all the social information, and we therefore define the social term as 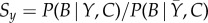. While this term encapsulates all the social information, it also depends on the non-social information *C*. An alternative parametrization that separates better the two types of information is possible but less compact, and leads to the same conclusions (electronic supplementary material, section S1). With these definitions, Bayes' theorem in equation (2.1) becomes2.2
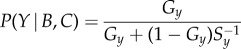
for option *y*, and similarly for option *x*. In our previous work, we developed the social terms *S*_*x*_ and *S*_*y*_, obtaining explicit forms that allow the model's predictions to be tested with experimental data [28,30,31]. This particularization is not necessary here because our results are independent of the exact form of the social terms. It suffices to note that *S*_*y*_ increases when social information indicates that *y* is a good option (usually as more individuals choose option *y*), and that *G*_*y*_ increases when private information indicates that *y* is a good option. By convention we will consider that *y* is the option chosen by the majority and hence favoured by social information, meaning that *S*_*y*_ > *S*_*x*_.

Decisions will be based on the estimated probabilities. A simple and widespread decision rule is to choose options according to the ratio of predicted qualities [28,30,32–41]. Then, the probability of choosing option *y* (*P*_*y*_) is a monotonically increasing function of the ratio of the estimated probabilities,2.3
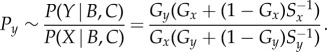
When *G*_*x*_ ≠ *G*_*y*_, private information will bias the decision towards one of the two options. This bias may be strong enough to obscure the effect of social information, and it is generally accepted that social information has more weight when the decision is most uncertain (i.e. when private information does not favour any option, *G*_*x*_ = *G*_*y*_) [42,43]. We will then focus our analysis on this state of maximum uncertainty, defining *G* ≡ *G*_*x*_ = *G*_*y*_. Then, equation (2.3) simplifies to2.4
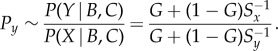
We now define adversity as an event that decreases the quality of all options. In our example, we may consider the case of a fire in the building. We assume that the deciding individual receives some information about the adverse event, such as a fire alarm going off. The fire alarm does not help to choose between the two doors (so we still have *G*_*x*_ = *G*_*y*_ ≡ *G*) but it lowers the estimated probability that either door leads safely to the street, because fire could be encountered on the way (so *G* decreases).

A simple assumption to explain super-aggregation in adversity is that social information becomes more relevant in this context, which in our model would mean a change in the social terms *S*_*x*_ and *S*_*y*_. But we aim to show that this change is not necessary for the result, so we will assume that these social terms remain constant (i.e. other individuals are equally reliable with and without the fire). Given that the social terms remain constant and that the fire alarm does not favour one door over another, should we expect super-aggregation around one of the options?

From equation (2.4) we see that, in extremely favourable conditions (*G* = 1), there will be no preference for either option. As adversity increases (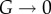), so does the preference for the majority option (figure 1*c*). Super-aggregation in adversity thus emerges, even when the social terms remain constant.

More formally, we say that super-aggregation in adversity occurs whenever the probability of choosing the majority option increases when lowering *G*, or equivalently,2.5
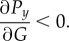
By computing the derivative of equation (2.4), we find that ∂*P*_*y*_/∂*G* has the same sign as (*S*^−1^_*y*_ − *S*^−1^_*x*_), which is negative as long as *y* is the majority option (hence *S*_*y*_ > *S*_*x*_). Therefore, the Bayesian model shows super-aggregation in adversity (see Material and methods for a more general derivation).

This derivation shows that agents following Bayesian estimations show super-aggregation in adversity in a two-option scenario with symmetric private information. We will next illustrate with an spatial example that this result can be generalized to many options and to asymmetric private information. Then we will discuss the mathematical properties that lead to super-aggregation in adversity, and discuss other models that also lead to it (outside Bayesian estimation and without using the ratio of the probabilities to estimate the tendency to choose either of them). Finally, we will contrast our predictions with existing experimental data.

### 2.1. Spatial super-aggregation in adversity

**Figure 2.**
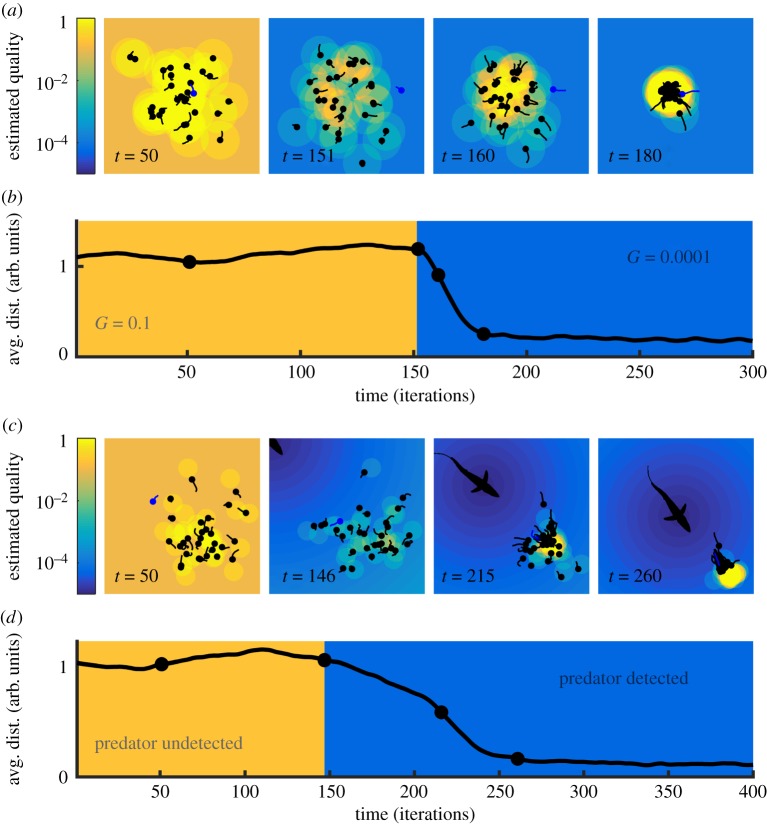
Spatial super-aggregation in adversity. (*a*) Four frames of a simulation where private information is identical for all locations and changes suddenly at time *t* = 150 from favourable conditions (*G* = 0.1) to adverse conditions (*G* = 0.0001). The background colour shows the probability that each pixel is a good location, estimated by the blue individual using the Bayesian model (see Material and methods). (*b*) Average distance among individuals as a function of time, for the same simulation as in (*a*). Background colour indicates the value of *G*. Dots mark the times of the frames shown on (*a*). (*c*) Same as (*a*), for a simulation with a predator. (*d*) Same as (*b*), for a simulation with a predator.

So far, we have seen that a Bayesian estimation model applied to a two-choice scenario shows super-aggregation in adversity. We now test that an extension of the same model to groups of moving animals gives the increased spatial cohesion typically observed in experiments. Our model draws from extensive work on collective motion [44–48] but, in contrast to most previous studies, our individuals do not react directly to each other. Instead, individuals estimate the quality of all points within a given maximum distance using a many-options version of the Bayesian model. In this model, social information increases the expected quality of the space around each individual, and individuals choose where to go with a probability proportional to each site's estimated quality (see Material and methods for details). In favourable conditions, a point close to other individuals has only slightly higher quality than the background, so the animals remain relatively dispersed. When conditions deteriorate, the estimated quality is high only for high-density regions, so all individuals converge towards them (figure 2*a*,*b*; electronic supplementary material, movies S1, S2).

The model also shows aggregation when the non-social estimate of qualities is asymmetric, as, for example, when a predator approaches the group. We assume that individuals who perceive the predator update their private information to a non-uniform map of qualities whose minimum is at the predator's position. A simple non-social model of predator avoidance in which each individual ran straight away from the predator would predict the group to disperse, its members following diverging trajectories centred on the predator's position. Likewise, in our model the gradient of privately estimated qualities pushes the individuals away from the predator. However, the gradient also decreases the overall quality, producing super-aggregation in adversity and leading to higher aggregation as a net result (figure 2*c*,*d*; electronic supplementary material, movie S3).

These results illustrate that decision-making can produce strong spatial super-aggregation in adversity under some conditions. Furthermore, as the effect emerges from the individuals' decision-making algorithm, the prediction is robust to changes in the dynamical details of the model, making it applicable across taxa (electronic supplementary material, figure S1).

### 2.2. A geometric framework for decision-making

We have seen super-aggregation in adversity in a Bayesian model, applied to a two-choice case and to a spatial version. We are still missing an explanation of the origin of the effect in this model and whether it applies more generally to other models. To answer these questions, we developed a simple framework in which many decision theories can be represented.

We divide the decision in two steps. First, the deciding individual estimates the quality of the available options (*Q*_*x*_ and *Q*_*y*_ for options *x* and *y*). In our Bayesian model, the quality of each option was the probability estimated by equation (2.1), *Q*_*y*_ = *P*(*Y* | *B*, *C*). In general, we will use ‘quality’ as a generic name for whatever is relevant to the decision, including both social and non-social factors. In the context of evolution, quality measures the fitness value of each option. In the language of economic decision-making, quality refers to the utility of each option. These qualities may be estimated with any model relevant for each particular situation.

**Figure 3.**
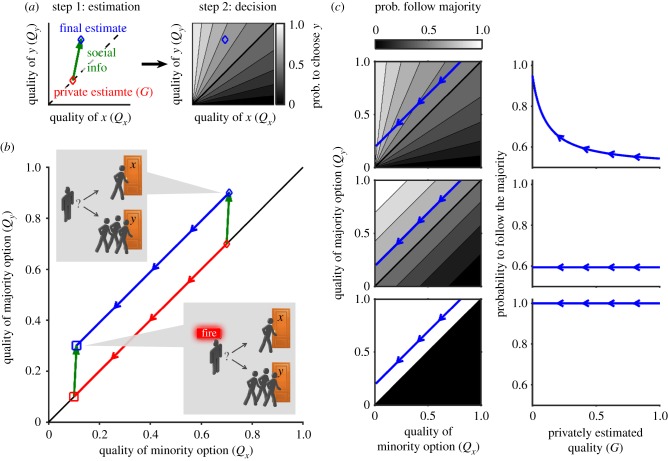
A geometric framework for decision-making. (*a*) Decision process: in step 1, social and non-social information are integrated to estimate the qualities of the options, *Q*_*x*_ and *Q*_*y*_. Red diamond: privately estimated qualities (*G*). Green arrow: contribution of the social information. Blue diamond: estimated qualities. In step 2, the decision rule gives the probability of choosing each option (*P*_*x*_ and *P*_*y*_) given the estimated qualities from step 1. In this example, *P*_*y*_ = *Q*_*y*_/(*Q*_*x*_ + *Q*_*y*_) and *P*_*x*_ = 1 − *P*_*y*_. (*b*) Example of the change in the estimated qualities when conditions become adverse. Colours as in (*a*). Diamonds: favourable conditions. Squares: adverse conditions. Blue and red arrowheads point in the direction of increasing adversity. (*c*) *Left*: Example of the trajectory followed by the estimated qualities in adversity (arrowheads point towards higher adversity), for three different decision rules: top: relative decision rule (*P*_*y*_ = *Q*_*y*_/(*Q*_*x*_ + *Q*_*y*_)); *middle*: absolute decision rule *P*_*y*_ = 1/(1 + e^−2(*Q*_*y*_^^−*Q*_*x*_^^)^); *bottom*: deterministic decision rule (*P*_*y*_ = 1 if *Q*_*y*_ > *Q*_*x*_, and *P*_*y*_ = 0 otherwise). *Right*: Probability to follow the majority as a function of the privately estimated quality, for the same trajectories and decision rules shown on the left.

For two options, we can visualize the estimated qualities in a two-dimensional landscape (figure 3*a*, left). We still assume symmetric private information (*G*_*x*_ = *G*_*y*_ ≡ *G*, where *G* now stands for the privately estimated quality), so the private estimate falls on the landscape's diagonal (figure 3*a*, red diamond symbol). Social information is then added (figure 3*a*, left, green arrow) to the private information to reach a final estimate (figure 3*a*, left, blue diamond symbol).

In the second step, a decision rule transforms the estimated qualities into the probability of choosing each option (figure 3*a*, right).

Now, we investigate the effect of adversity on the decision-making process. As before, the effect of adversity is to lower the privately estimated quality *G*, moving the estimation to a different point of the quality landscape. To illustrate this, we may consider an estimation rule simpler than our Bayesian model: we define the quality of an option as *Q*_*y*_ = *G*_*y*_ + *S*_*y*_, where the social term (*S*_*y*_) remains constant in adversity (figure 3*b*). Even in this linear case, and even if the change in non-social information does not favour one option over the other, the change in estimated qualities leads in general to a different probability of choosing each option (figure 3*c*, top). The only exception is when the estimated qualities run along an isoprobability line of the decision rule (figure 3*c*, middle). Such a perfect match is unlikely except for decision rules with large regions of constant probability, as, for example, the deterministic rule ‘always choose the option with highest estimated quality’ (figure 3*c*, bottom). This decision rule is used in many simple models, preventing them from explaining super-aggregation in adversity [26,27].

Another feature that prevents some probabilistic models from predicting super-aggregation in adversity is that they assume mutually excluding options (one of them is good and the other is bad, so *P*(*x* is good) = 1 − *P*(*y* is good)) [26–28]. These models lack a parameter that measures adversity, which would require lowering *P*(*x* is good) and *P*(*y* is good) simultaneously, and cannot predict super-aggregation in adversity.

In general, decision rules are not deterministic and options are not mutually excluding, so the probability of following the majority will change with adversity. But why do we usually observe increased (rather than decreased) aggregation in adversity? The geometric framework does not directly answer this question, but will help us visualize how super-aggregation in adversity emerges from different characteristics common to many decision-making models.

### 2.3. Mechanisms for super-aggregation in adversity

This section will discuss several qualitative mechanisms that lead to super-aggregation in adversity. The mechanism considered in most existing models to describe super-aggregation in adversity [6,20–25] amounts to an explicit change in the social terms that favours the majority option. This change is probably present in many situations, and it is easy to see that it would change the degree of aggregation. Here we will focus on another three mechanisms that are present even when the social terms remain constant.

#### 2.3.1. Relative decision rules

Many decisions depend on the relative value of estimated qualities, rather than on their absolute value. Examples range from bacteria to humans, including rules such as Weber's Law (which depends on the relative difference between qualities (*Q*_*y*_ − *Q*_*x*_)/(*Q*_*y*_ + *Q*_*x*_)), probability matching (which depends on *Q*_*y*_/(*Q*_*x*_ + *Q*_*y*_)), direct ratio rules (*Q*_*y*_/*Q*_*x*_) and others [28,30,32–41]. All these rules share the condition that2.6
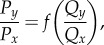
where *P*_*x*_ and *P*_*y*_ are the probabilities of choosing *x* and *y*, respectively, and *f* is any monotonically increasing function. When more than two options exist, a relative decision rule must fulfil equation (2.6) for every pair of options.

**Figure 4.**
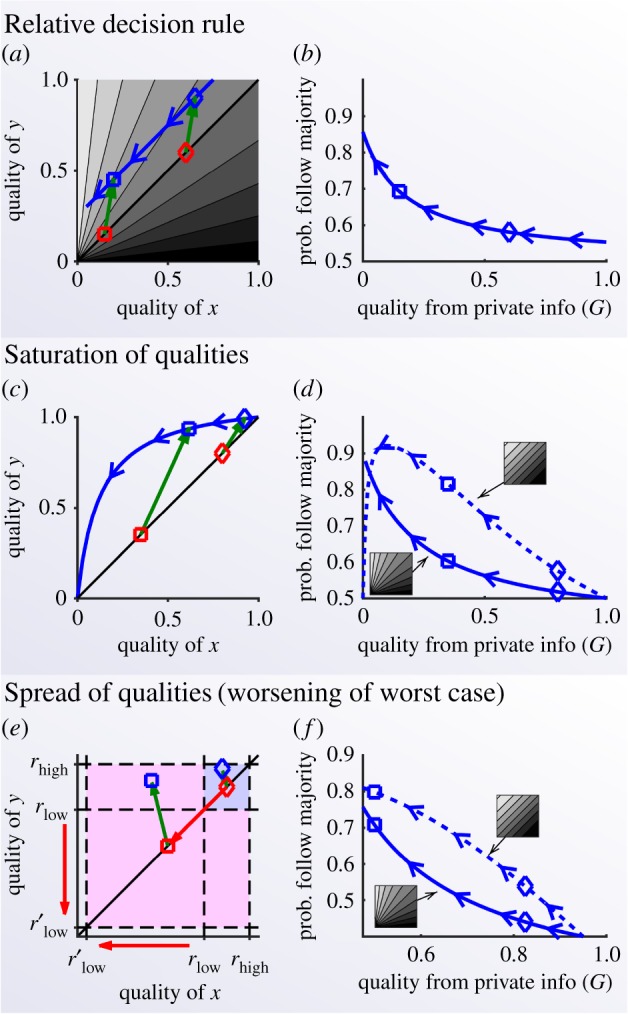
Mechanisms responsible for super-aggregation in adversity. (*a*) Estimated qualities for an additive estimation model (*Q*_*x*_ = *G* + *S*_*x*_, *Q*_*y*_ = *G* + *S*_*y*_, with *S*_*x*_ = 0.05, *S*_*y*_ = 0.3). Red: private estimate for favourable conditions (*G* = 0.6, diamond) and adverse conditions (*G* = 0.15, square). Green arrows: contribution of the social information. Blue: trajectory of the final estimated qualities when adversity increases (arrows point towards adversity). Background: probability of choosing *y* for each pair of qualities, for the relative decision rule *P*_*y*_ = *Q*_*y*_/(*Q*_*x*_ + *Q*_*y*_) (greyscale: black=0, white=1). (*b*) For the model in (*a*), probability of choosing the majority option (*y*), as a function of the privately estimated quality (*G*). Arrows point in the direction of adversity. Diamond and square correspond to the same conditions as in (*a*). (*c*) Estimated qualities for a Bayesian decision-making model (equation (2.2) with *S*_*x*_ = 3, *S*_*y*_ = 9). Colours and symbols as in (*a*). (*d*) Probability of choosing the majority option (*y*) as a function of the privately estimated quality (*G*), using the Bayesian estimation model in (*c*) and a relative decision rule (*P*_*y*_ = *Q*_*y*_/(*Q*_*x*_ + *Q*_*y*_), solid line) or an absolute decision rule (*P*_*y*_ = 1/(1 + e^−2(*Q*_*y*_^^−^^*Q*_*x*_^^)^), dashed line). (*e*) Estimated qualities for the model with pay-offs (equation (4.17)); *r*_high_ is the pay-off for a good option, *r*_low_ the pay-off for a bad option in favourable conditions and *r*′_low_ the pay-off for a bad option in adverse conditions. Colours and symbols as in (*a*). (*f*) Same as (*d*) but for the model in (*e*). Quality from private info is defined as *G* = *r*_low_ + (*r*_high_ − *r*_low_)*P*_private_, where *P*_private_ = 0.5 is the privately estimated probability that options have a high reward.

All relative rules have isoprobability lines identical in shape to those in figure 4*a*, independently of function *f*. With these rules, when the effect of social information on the estimation remains the same (figure 4*a*, green arrows), its impact on the final decision increases in adversity, with a higher probability of following the majority (figure 4*b*). Even when the influence of social information in the qualities decreases in adversity, the probability to follow the majority will increase as long as the estimated qualities fulfil2.7
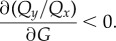
This condition holds both for the non-optimal estimation rule in figure 4*a* and for the Bayesian model in equation (2.1) (as illustrated in figure 1*c* and derived in equation (4.6) in Material and methods).

#### 2.3.2. Saturation near the upper bound of qualities

The range of qualities is often bounded, for example, when there is a limit in the amount of food an animal can consume or when qualities are probabilities. In this case, as the privately estimated qualities increase, both the private and the final estimates converge to the upper bound, so the difference between them—the contribution of social information—tends to zero. Adversity lowers the qualities and therefore takes estimation away from the upper bound, and social information can now impact more. This effect takes place in the Bayesian model in equation (2.2), because the qualities are defined as probabilities (figure 4*c*). Therefore, the Bayesian model may show super-aggregation in adversity not only when coupled with a relative decision rule, but also through the saturation near the upper bound.

To decouple the effects of saturation and relative decision rules, we considered a decision rule that depends on the difference of the qualities, *Q*_*y*_ − *Q*_*x*_ (Material and methods). With this decision rule, Bayesian estimation predicts super-aggregation in adversity when the initial estimated qualities are high (dashed line in figure 4*d*). In the range of very low qualities we find the opposite effect, due to the lower bound of the probability. However, social information usually pushes the estimation towards the upper bound, making the range for super-aggregation in adversity wider than the range for the opposite effect, especially for large groups (equation (4.16) in Material and methods).

#### 2.3.3. Spread of the qualities through worsening of the worst-case scenario

A third mechanism was found for the case in which adversity modifies the values of the potential pay-offs behind each option, rather than the probability of reaching them. In our example of two doors, we defined the quality as the probability to reach the street safely. We may build a more detailed model by detaching the probability that each door reaches the street and its consequences (the pay-off behind each option). A fire alarm does not change the probability that each door leads to the street, nor the corresponding pay-off for reaching the street (*r*_high_). Instead, it decreases the pay-off for choosing the wrong door from a low value *r*_low_ (detour to street) to an even lower pay-off *r*′_low_ < *r*_low_ (possibility of dying in a fire). Adversity thus increases the contrast between a good and a bad choice, effectively rescaling the estimation from the region between *r*_low_ and *r*_high_ to a wider region of the quality landscape between *r*′_low_ and *r*_high_ (figure 4*e*). This rescaling results in a larger contribution of the social information, leading to super-aggregation in adversity for both relative and absolute decision rules (figure 4*f*) and for all combinations of the parameters (Material and methods).

The opposite effect takes place when adversity affects the gains of a good option, rather than the cost of a bad one (Material and methods). Therefore, among adverse situations, we expect the life-threatening ones—characterized by a deterioration of the worst-case scenario—to produce stronger super-aggregation in adversity.

Each of these three mechanisms can produce super-aggregation in adversity by itself, but in general we may observe several of them reinforcing each other, as, for example, the solid lines in figure 4*d*,*f* .

### 2.4. Contrast with experimental data

**Figure 5.**
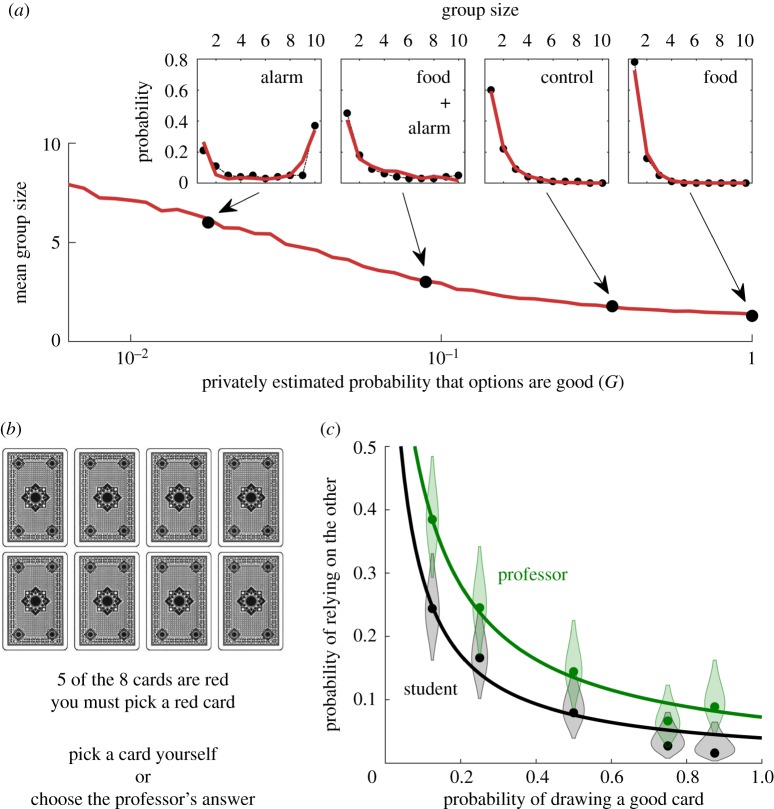
Experiments are consistent with super-aggregation in adversity emerging from decision-making. (*a*) Mean group size for 10 fish swimming in a closed space as a function of the privately estimated quality, as predicted by the spatial model. The black dots indicate the experimental mean group sizes [7], and the values of *G* that best fit the experiments. Insets: distributions of group sizes for the four experiments (dots: experimental data. red line: simulations). (*b*) Layout of the experiment of Case *et al.* [49]. (*c*) Probability of relying on the card choice of another person (a professor for the green data and a student for the black data), as a function of the proportion of red cards (which is equivalent to the probability of getting a good card at random). Dots: experimental data (curved patches indicate experimental uncertainty due to sampling, with width proportional to the probability that the true value falls at each value, and truncated at the 95% CI). Lines: model (equation (4.24)).

We have tested whether decision models showing super-aggregation in adversity compare well against experimental data. We compared the spatial model shown in figure 2 with experimental data of groups of 10 fish (*Fundulus diaphanus*) [7]. To modify the private information of the fish, the authors sprayed different odours uniformly on the water. Food odour was used to signal favourable conditions, and alarm odour (released when a fish is killed by a predator) to signal adversity. Experimenters then measured the distribution of group sizes in each treatment. They found that odours that signal adversity increased the cohesion in the fish, measured as a higher probability of finding large groups (figure 5*a*, insets). Our spatial model reproduces these data with an accuracy comparable to that of the model originally proposed to describe them [7]. Our approach has the advantage that the theory indicates what parameter must change across conditions, while in previous models the increase in social attraction could be implemented in several different ways. In our model, all parameters remain constant across conditions except the private information *G*, which we fitted to the data (figure 5*a*; see Material and methods for details).

These results are too indirect to conclude that fish are effectively implementing a Bayesian model with the values of *G* recovered by our fit. However, they show that our prediction for super-aggregation in adversity can be strong enough to reproduce experimental results.

Super-aggregation in adversity may also take place in humans, as suggested by the occurrence of sudden bank runs [10–12] and human stampedes [13–19]. It is, however, unclear whether these events actually emerge from super-aggregation, from other causes such as sudden changes in the private information towards a single preferred option, or from a combination of both. Tests in these panic situations are difficult, but our theory suggests that super-aggregation in adversity should also happen when adversity is unrelated to life-threatening situations. In these situations our prediction is easy to test, and we did so using an existing dataset [49].

In this study, the experimental subject had to choose one out of eight face-down cards and got a reward when the chosen card was red. The subject could either choose one card or rely on the opinion of another person—either a professor or a student (figure 5*b*). Subjects were told the proportion of red cards, so they had private information about the probability of success. For this dataset, the experimenters expected no relation between this probability of success and the probability to rely on the other person's opinion [49]. However, the data show the trend predicted by our theory: when subjects rely more on social information the proportion of good cards is lower (figure 5*c*; Material and methods).

## 3. Discussion

Our main result is that estimations and decisions naturally imply, without extra hypotheses, super-aggregation in adversity. It is, therefore, a parsimonious explanation for the generality of super-aggregation in adversity, which should be common to all situations in which estimation plays a role. This is consistent with the general presence of super-aggregation in adversity [1–5,7], but of course does not rule out additional mechanisms in particular situations, such as collective protection or confusion effects in the case of predation risk. Interestingly, our approach also makes predictions that are probably different from other explanations. This is more dramatically seen in those cases in which our approach ceases to predict super-aggregation in adversity. This happens for very adverse situations when the decision rule is not relative (figure 4*d*). Also, we predict the opposite effect, a form of dispersion in adversity, in the case in which a higher pay-off decreases.

Our results are complementary to other mechanisms that may increase aggregation in adversity in specific situations [20–23], and in fact our geometric framework may naturally include some of these models. For example, it can reproduce the selfish-herd hypothesis [21] when the qualities are given by the probability of surviving a predator attack (electronic supplementary material, figure S2). In general, it is difficult to disentangle different mechanisms in a given situation. For example, the aggregation typically found when animals explore a new set-up can also be due to increased uncertainty in private information; the decreased aggregation found experimentally as a response to food odour may be a mechanism to reduce competition. Our results add a new potential mechanism, to be considered along with the classical ones.

Our results apply to the case when the presence of other individuals in one particular option increases its expected quality (i.e. we assume that *S*_*y*_ > *S*_*x*_ when *y* is the majority option). This may not be the case when social information is communicated through other means, such as by how insistently an individual prefers one option. In this case, the minority may drive the group towards a given option [50,51] and, while the amplification of the effect of social information will still exist, it may not lead to observable super-aggregation. Super-aggregation in adversity may also not exist if competition is strong, outweighing the benefits of social information. In this case, we might expect different—perhaps even opposite—trends. Some situations may be mixed, with super-aggregation in adversity leading to high density around one of the options, in turn leading to high competition. For example, super-aggregation in adversity may lead to most people choosing the same emergency exit in a building, producing increased competition for space when the crowd reaches the door and obstructs it.

Our results may have implications on the neural architectures behind optimal decisions. Optimal decisions leading to super-aggregation in adversity may not require recomputing the social terms *S*_*x*_ and *S*_*y*_, opening the possibility of neural systems in which social and non-social information are computed by separate modules, but that can nevertheless combine them optimally and efficiently. This would allow decision-makers to swiftly adapt their social behaviour to adverse conditions. Even in situations in which social information changes substantially, a first-order approximation that would emerge from this architecture would be to update the non-social component and recompute the final decision before updating the social component. Therefore, the additional constraint of making fast decisions preserves super-aggregation in adversity.

Our analysis has focused on symmetric private information (all options have the same privately estimated quality), which corresponds to maximum uncertainty about what option is better. The mechanisms discussed are also at work with asymmetric private information, but in this case it is difficult to decouple the role of private and social information. An additional confounder arises if the asymmetry in private information between the options increases or decreases in adversity, obscuring the effect of social information. For example, many situations are characterized by a sharp increase in asymmetry: people wandering in a building usually have different aims, but a fire alarm will direct everyone towards the exit. This type of aggregation is compatible with our results and fits in our framework, but our results are applicable even when considering symmetric options, as, for example, two identical emergency exits or two equivalent escape routes from a predator. In general, by sticking to the symmetric case, we have shown that super-aggregation in adversity does not require any asymmetry in the private information.

We have seen that super-aggregation in adversity is at the core of decision-making. This finding does not exclude other causes for the observed behaviours, but may be taken as a reference result for decision-making before resorting to more complex explanations.

## 4. Material and methods

### 4.1. Formal definition of super-aggregation in adversity

For a given decision-making model, we will say that super-aggregation in adversity occurs whenever4.1
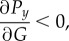
where *P*_*y*_ is the probability to choose the majority option and *G* is the privately estimated quality (equal for all options because we consider symmetric private information).

### 4.2. Super-aggregation in adversity with relative decision rules

We start from the definition of super-aggregation in adversity (equation (4.1)). If there are only two options (*x* and *y*), we must have *P*_*x*_ = 1 − *P*_*y*_. Therefore, decreasing *P*_*y*_ necessarily means decreasing *P*_*y*_/*P*_*x*_, so4.2
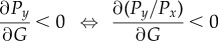
and from equation (2.6) we have that4.3
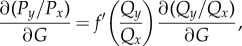
where *f*′( · ) is the derivative of *f* with respect to its argument, which is always positive because *f* is by definition a monotonically increasing function. Therefore,4.4
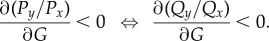
Putting together equations (4.2) and (4.4), super-aggregation in adversity will take place whenever4.5
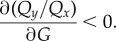


If the choice is among more than two options, a similar condition is sufficient (but not necessary) to produce super-aggregation in adversity. The probability to choose the majority option must decrease if the ratio of this probability with respect to all other probabilities decreases:4.6
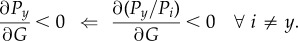
With this and equation (4.4) (which is valid for any two options), we have that4.7
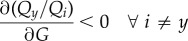
is a sufficient condition for super-aggregation in adversity when there are more than two options.

For the Bayesian model in equation (2.1), *Q*_*y*_/*Q*_*x*_ is given by equation (2.4). Therefore,4.8
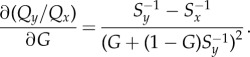
This expression is always negative because squared terms are always positive and *S*_*y*_ > *S*_*x*_ because *y* is the majority option. Therefore, the Bayesian estimation model with a relative decision rule always produces super-aggregation in adversity.

### 4.3. Absolute decision rules

We say that a decision rule is absolute when the probability to choose option *y* is of the form4.9
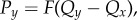
where *Q*_*x*_, *Q*_*y*_ are the qualities of options *x* and *y*, and *F* is any monotonically increasing function.

Absolute decision rules give super-aggregation in adversity whenever equation (4.1) is fulfilled. From equation (4.9),4.10
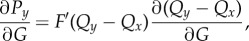
where *F*′( · ) is the derivative of *F* with respect to its argument, which is always positive because *F* is monotonically increasing. Therefore, ∂*P*_*y*_/∂*G* < 0 ⇔ ∂(*Q*_*y*_ − *Q*_*x*_)/∂*G* < 0, so super-aggregation in adversity will take place whenever4.11
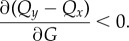


Absolute decision rules may emerge, for example, if the subject chooses *y* when *Q*_*y*_ > *Q*_*x*_ + *η*, with *η* a random number: let *ρ*(*η*) be the probability density function of *η*, and *F*(*η*) be its cumulative distribution. Then, the probability to choose *y* is equal to the probability to draw a value of *η* lower than *Q*_*y*_ − *Q*_*x*_,4.12
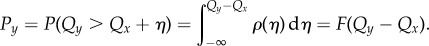
We see that *F* is a cumulative distribution, which is always monotonically increasing. In figures 1 and 4, we have used4.13
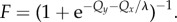
This function is the cumulative distribution of the logistic distribution, whose probability density function is *ρ*(*η*) = e^−*η*/λ^λ^−1^(1 + e^−*η*/λ^)^−2^. We have chosen this distribution because its cumulative density function has a simple analytical form; all results hold regardless of the probability distribution that we choose.

When combined with an absolute decision rule, the Bayesian model will show super-aggregation in adversity when4.14
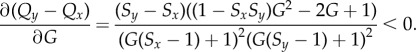
The denominator of this expression is always positive, and *S*_*y*_ − *S*_*x*_ is positive when *y* is the majority option. Therefore, the whole expression is negative when4.15
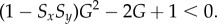
This inequality has only one solution for *G* > 0, which is4.16
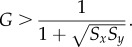
Thus, there is super-aggregation in adversity in the range of high *G*, and the opposite effect in the range of low *G*. The higher the *S*_*x*_*S*_*y*_, the wider the range for super-aggregation in adversity.

This result matches the intuition that the upper bound is more important than the lower one because social information tends to move the estimation towards the upper bound. In principle, an individual choosing option *x* can indicate both that *x* is a good option and/or that *y* is a bad one. Therefore, this choice can increase *S*_*x*_ to the same degree as it decreases *S*_*y*_. However, experimental data show that social information usually has a positive net effect, meaning that an individual that chooses *x* increases *S*_*x*_ more than it decreases *S*_*y*_ (in our previous work [30] we defined parameter *k* to measure this effect. All experimental data, from three different species, were consistent with *k* < 1, meaning that social information has a positive net effect). When social information has a positive net effect, the product *S*_*x*_*S*_*y*_ increases as more individuals make choices. Therefore, for large groups (or when behaviours are very informative) the range of super-aggregation in adversity is very wide. For example, in our previous study [30] we found that for zebrafish *S*_*x*_*S*_*y*_ = 5^*n*^, where *n* is the total number of individuals that have already chosen one of the two options. Therefore, equation (4.16) tells us that a group of 10 zebrafish will show super-aggregation in adversity for *G* > 3 × 10^−4^, and a group of 15 for *G* > 6 × 10^−6^. As a reference, in our experiments (that corresponded to an intermediate level of adversity, with the fish in an unknown environment but without any direct threat) we found *G* ≈ 0.08 [30].

### 4.4. Model with pay-offs

Let *r*_low_ be the reward provided by a bad option, and *r*_high_ be the reward provided by a good one, with *r*_high_ ≥ *r*_low_ ≥ 0. The estimated quality of option *x* is its expected pay-off4.17
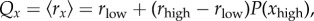
and similarly for option *y*. By *P*(*x*_high_) we denote the estimated probability that *x* has a high pay-off (*r*_high_), and equation (4.17) takes into account that *P*(*x*_low_) = 1 − *P*(*x*_high_). The private estimate of the quality of the options is4.18
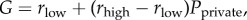
where *P*_private_ is the privately estimated probability that an option contains a high reward (equal for all options, by hypothesis). A change in private information may translate into three different changes in the parameters: (i) a change in the probability (*P*_private_), (ii) a change in the lower pay-off (*r*_low_) and (iii) a change in the higher pay-off (*r*_high_). The first type of change will depend on the specific model we use to estimate the probabilities (we may use a Bayesian model as in previous sections, or any other model). Here we will consider the other two cases, in which the new private information changes the values of the rewards that the subject expects to obtain.

First, let us consider the case in which adversity means a decrease in the lower reward (*r*_low_), and the case of a relative decision rule (equation (2.6)). From equation (4.18) we have that ∂*G*/∂*r*_low_ > 0 in all cases. Therefore, any derivative with respect to *G* will have the same sign as a derivative with respect to *r*_low_, and equation (2.7) is now equivalent to ∂(*Q*_*y*_/*Q*_*x*_)/∂*r*_low_ < 0. We compute this derivative from equation (4.17), getting4.19
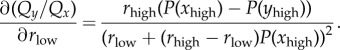
The denominator is always positive because it is squared, and *r*_high_ is positive by definition. We see that *P*(*y*_high_) > *P*(*x*_high_) because *y* is the majority option, so the derivative is always negative; super-aggregation in adversity always takes place.

We find the same result for an absolute decision rule. To test for super-aggregation in adversity, we use equation (4.11), deriving with respect to *r*_low_ instead of *G* (as explained in the previous paragraph). We get4.20
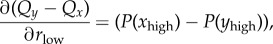
which is always negative because *P*(*y*_high_) > *P*(*x*_high_) when *y* is the majority option. Therefore, cohesion always increases with adversity.

By contrast, cohesion decreases in adversity when the higher pay-off changes, keeping all other parameters constant. This has a simple intuitive explanation: in adversity *r*_high_ goes down, becoming more similar to *r*_low_. Therefore, the difference between choosing correctly and choosing incorrectly decreases, making any decision weaker. Mathematically, for relative decision rules we evaluate the condition in equation (2.7) (in this case deriving with respect to *r*_high_ instead of *G*), finding4.21
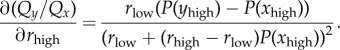
The denominator of this expression is always positive, as is *r*_low_ by definition. And *P*(*y*_high_) > *P*(*x*_high_) because *y* is the majority option, so the derivative is always positive, meaning that cohesion decreases in adversity when using a relative decision rule. Now we consider an absolute decision rule (equation (4.11)):4.22
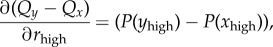
which is always positive because *P*_*y*_ > *P*_*x*_ when *y* is the majority option.

### 4.5. Spatial model

To simulate animals in motion, we discretize the space in pixels (for two-dimensional simulations) or voxels (for three-dimensional simulations). In each iteration, each individual chooses one pixel and accelerates towards it, up to a maximum acceleration (*a*_max_) and never exceeding a maximum velocity (*v*_max_). The probability to choose a given pixel is proportional to the probability that it is a good place according to the Bayesian model in equation (2.1). Following Arganda *et al.* [30], we expand the social term as *S*_*y*_ = *s*^*N*_near_^, where *y* here refers to a single pixel (or voxel), *N*_near_ is the number of individuals within a radius *r*_influence_ from pixel *y* and *s* is a parameter measuring the reliability of the other individuals. We, therefore, have4.23
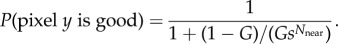
We also incorporate a limited field of view for each individual, by assuming that an individual can only choose pixels within a certain radius from itself, *r*_view_ (this parameter is needed to make the model computationally tractable even when the individuals are not restricted to a finite region).

For the results in figure 3*a*,*b* and electronic supplementary material, movie S1, we chose the parameters *a*_max_ = 0.1, *v*_max_ = 0.5, *r*_influence_ = 5, *r*_view_ = 100, *s* = 3 and *G* as indicated in figure 3*b* (space units are in pixels, and time units in iterations).

For the case of predator avoidance the model confirmed our prediction of increased cohesion in adversity, but was not realistic: in many conditions, when the predator appears, individuals cluster together but fail to run away from it (the social attraction overcomes the repulsion from the predator). To obtain more realistic results, we assume that an individual indicates that the location towards which it is heading (rather than its current location) is a good place. To account for this, we centre the circle of influence of each individual at the position where it will be in *t*_prediction_ steps in the future (assuming it will keep constant direction and speed), rather than at its current location. With this addition, animals not only cluster together when the predator appears, but also tend to align with each other along the optimal escape route (figure 3c,d; electronic supplementary material, movie S2; simulations with the same parameters as in previous paragraph, but with *t*_prediction_ = 15).

In fact, alignment arises in adversity when individuals pay attention to future positions rather than current ones (*t*_prediction_ > 0), even if there is no predator present (electronic supplementary material, movie S3; simulations with same parameters as for electronic supplementary material, movie S1, but with *t*_prediction_ = 15). Thus, in our model the group does not need the asymmetry created by the predator to reach a consensus direction. But if there is a predator, the consensus direction will head away from it.

To reproduce the experimental data in figure 5*a*, we adjusted the model parameters to mimic the experimental conditions [7]. The experiments were performed in a 100 × 100 × 10 cm tank, and the experimenters considered that two fish belonged to the same group when they were closer than 16 cm. Assuming a scale of 1 pixel cm^−1^, we simulated 10 individuals in a closed two-dimensional space of 100 × 100 pixels and set *r*_influence_ = 16 pixels. When performing the analysis, we considered that two fish belonged to the same group when they were within 16 pixels. Then we searched the parameter space manually and non-systematically, finding a good fit for *a*_max_ = 1, *v*_max_ = 12, *r*_view_ = 30, *s* = 10, *t*_prediction_ = 0 and *G* as in figure 4*a*.

### 4.6. Model for the cards experiment

The experimental design presented social information as a separate option (figure 5*b*), so we have implemented the simplest model that mimics this condition: the subject assumes that the other person (the professor or the student) has a fixed probability of making a correct choice (*ξ*). And the probability of making a correct choice by choosing a card is *n*_red_/8, where *n*_red_ is the number of red cards. We then consider a nine-choice scenario: the eight cards plus the social option. As the decision rule we use probability matching, where the probability to choose one option is proportional to its corresponding probability of success. Then, the probability to rely on the other's opinion is4.24
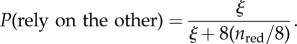
We fit the parameter *ξ* to the experimental data by minimizing the sum of the squared errors between the data and the model, getting *ξ* = 0.624 for the professor and *ξ* = 0.328 for the student.

## Supplementary Material

Supplementary Material

## Data accessibility

Experimental datasets used were obtained from Hoare *et al.* [7] and Case *et al.* [49].
