# Supplementary material for: Adversity magnifies the importance of social information in decision-making
Source: J R Soc Interface. 2017 Nov 29;14(136):20170748. doi: 10.1098/rsif.2017.0748 (PMC5721171; doi:10.1098/rsif.2017.0748)
Supplement: Supplementary Material [file rsif20170748supp1.pdf]

# SUPPLEMENTARY MATERIAL: Adversity magnifies the importance of social information in decision-making

Alfonso Pérez-Escudero* & Gonzalo G. de Polavieja†

## 1 Alternative Bayesian model

The Bayesian model used in the main text assumes that the quality of each option is estimated independently of the other. Here we remove this approximation, arriving to a different and more complex model. Regarding superaggregation in adversity, we find the same conclusions as for the simpler model presented in the main text.

### 1.1 Derivation of the estimated qualities

Let us consider a choice between two options, $x$ and $y$. Each option can be good or bad; we will write $X$ to denote '$x$ is good' and $\bar{X}$ to denote '$x$ is bad', and similarly for option $y$. The pair of options can be in four possible states: both options are good ($XY$), both options are bad ($\bar{X}\bar{Y}$), or one option is good and the other is bad ($X\bar{Y}$ and $\bar{X}Y$). We calculate the probability for each of these states, using both private information ($C$) and the behaviours of the other individuals ($B$). For example, the probability that option $x$ is good and option $y$ is bad, using Bayes', theorem, is

$$P(X\bar{Y}|B,C) = \frac{P(B|X\bar{Y},C)P(X\bar{Y}|C)}{\Omega}, \tag{S1}$$

where

$$\Omega = P(B|XY,C)P(XY|C) + P(B|X\bar{Y},C)P(X\bar{Y}|C) + \\ + P(B|\bar{X}Y,C)P(\bar{X}Y|C) + P(B|\bar{X}\bar{Y},C)P(\bar{X}\bar{Y}|C). \tag{S2}$$

The term $P(X\bar{Y}|C)$ contains the private information about the state of both options, and the term $P(B|X\bar{Y},C)$ contains the social information. If we assume that the two

*perezesc@mit.edu – Physics of Living Systems, Physics Department, MIT
†gonzalo.polavieja@neuro.fchampalimaud – Champalimaud Neuroscience Programme, Champalimaud Center for the Unknown

options can be good or bad independently, we have $P(X\bar{Y}|C) = P(X|C)P(\bar{Y}|C)$. Now we define $G_x = P(X|C)$ and $G_y = P(Y|C)$, so $P(X\bar{Y}|C) = G_x(1 - G_y)$. If we further assume that private information is symmetrical ($G_x = G_y \equiv G$), Equation S1 becomes

$$P(X\bar{Y}|B,C) = \frac{P(B|X\bar{Y},C)G(1-G)}{P(B|XY,C)G^2 + [P(B|X\bar{Y},C) + P(B|\bar{X}Y,C)]G(1-G) + P(B|\bar{X}\bar{Y},C)(1-G)^2}. \tag{S3}$$

The four probabilities $P(B|XY,C)$, $P(B|X\bar{Y},C)$, $P(B|\bar{X}Y,C)$ and $P(B|\bar{X}\bar{Y},C)$ parametrize the available social information. Because they must sum one, we only have three free parameters. It is therefore more useful to define $S_\mathrm{b} \equiv P(B|XY,C)/P(B|\bar{X}\bar{Y},C)$, $S_x \equiv P(B|X\bar{Y},C)/P(B|\bar{X}\bar{Y},C)$ and $S_y \equiv P(B|\bar{X}Y,C)/P(B|\bar{X}\bar{Y},C)$, and write Equation S3 as

$$P(X\bar{Y}|B,C) = \frac{S_x G(1-G)}{S_\mathrm{b}G^2 + (S_x + S_y)G(1-G) + (1-G)^2}. \tag{S4}$$

The probabilities for the other three states can be derived in the same way:

$$P(\bar{X}Y|B,C) = \frac{S_y G(1-G)}{S_\mathrm{b}G^2 + (S_x + S_y)G(1-G) + (1-G)^2} \tag{S5}$$

$$P(XY|B,C) = \frac{S_\mathrm{b}G^2}{S_\mathrm{b}G^2 + (S_x + S_y)G(1-G) + (1-G)^2} \tag{S6}$$

$$P(\bar{X}\bar{Y}|B,C) = \frac{(1-G)^2}{S_\mathrm{b}G^2 + (S_x + S_y)G(1-G) + (1-G)^2}. \tag{S7}$$

Now we define the quality of $x$ as the probability that $x$ is good (and the same for option $y$), getting

$$Q_x = P(X|B,C) = P(XY|B,C) + P(X\bar{Y}|B,C) = = \frac{S_\mathrm{b}G^2 + S_x G(1-G)}{S_\mathrm{b}G^2 + (S_x + S_y)G(1-G) + (1-G)^2} \tag{S8}$$

$$Q_y = P(Y|B,C) = P(XY|B,C) + P(\bar{X}Y|B,C) = = \frac{S_\mathrm{b}G^2 + S_y G(1-G)}{S_\mathrm{b}G^2 + (S_x + S_y)G(1-G) + (1-G)^2}. \tag{S9}$$

## 1.2 Effect of a relative decision rule

We assume that $y$ is the majority option. If the decision rule is relative (Equation 3 of the main text), superaggregation in adversity will take place when $\frac{\partial(Q_y/Q_x)}{\partial G} < 0$ (Equation 7 of the main text). From Equations S8 and S9,

$$\frac{\partial(Q_y/Q_x)}{\partial G} = \frac{S_\mathrm{b}(S_x - S_y)}{[G(s_b - S_x) + S_x]^2}. \tag{S10}$$

The denominator of this expression is always positive because it is squared. $S_\mathrm{b}$ is always positive because it is a ratio of probabilities. And $S_y > S_x$ because $y$ is the majority option, so this derivative is always negative. Therefore, there is superaggregation in adversity for any values of the parameters.

### 1.3 Effect of an absolute decision rule

If the decision rule is absolute (Equation 10 in the main text), superaggregation in adversity will take place when $\frac{\partial(Q_y - Q_x)}{\partial G} < 0$ (Equation 12 of the main text). From Equations S8 and S9,

$$\frac{\partial(Q_y - Q_x)}{\partial G} = \frac{(S_x - S_y)[(s_b - 1)G^2 + 2G - 1]}{[S_\mathrm{b}G^2 + (S_x + S_y)G(1 - G) + (1 - G)^2]^2}. \tag{S11}$$

The denominator is always positive because it is squared. $S_x - S_y$ is always negative because $S_y > S_x$ when $y$ is the majority option. Therefore, the sign depends on the sign of $(s_b - 1)G^2 + 2G - 1$. This polynomial has a single root between 0 and 1 at $G = (\sqrt{s_b}+1)^{-1}$. Therefore, the derivative is negative when $G > (\sqrt{s_b}+1)^{-1}$, recovering the same result as for the Bayesian model in the main text: there is superaggregation in adversity in the regime of high $G$, and the opposite effect in the regime of low $G$.

## 2 Superaggregation in space emerges for a wide range of dynamical parameters

The emergence of superaggregation in adversity in our spatial model does not depend on a particular choice of dynamical parameters. To illustrate this, we have run simulations both in 2D and 3D, and with random parameters of the dynamical model (speed, acceleration, etc). Superaggregation in adversity arises in most cases, independently of these details (Figure S1e).

## 3 The selfish herd hypothesis in the quality landscape

We assume that the available space is divided in $M$ possible locations. The $i$-th location is occupied by $n_i$ individuals ($i = 1 \dots M$). The quantities $n_i$ do not count the focal individual, which starts from any given location. A predator may arrive to any location with probability $1 - G$ (we define it in this way to keep the convention that $G$ decreases when conditions become adverse). If the predator arrives, it will eat one of the individuals in that location, chosen at random. We define the quality of each option as the probability that the focal individual survives after choosing that location, so for location $k$ we have

$$Q_k = P(\text{survive in location } k) = 1 - \frac{1 - G}{(n_k + 1)}, \tag{S12}$$

where $n_k + 1$ is the number of individuals in option $k$, assuming that the focal individual chooses it and that no other individual moves in the current round.

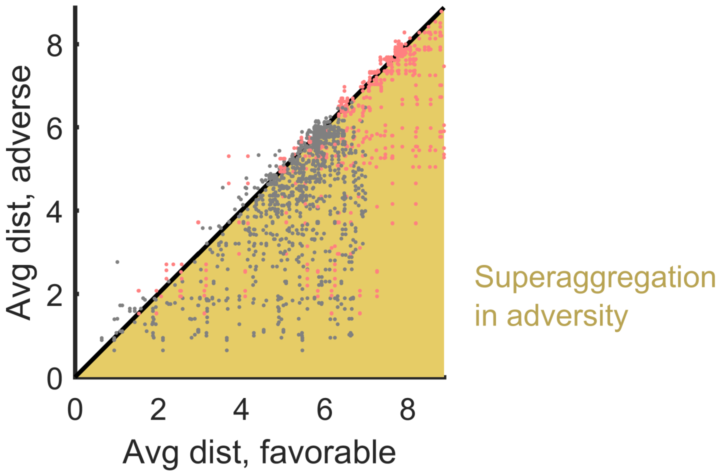


Figure S1: Average distance between 10 individuals when conditions are adverse (low $G$), vs. their average distance when conditions are favorable (higher $G$; points below the diagonal indicate superaggregation in adversity). Each average distance comes from 50 simulations with identical parameters and random initial positions. Each simulation lasts 200 iterations. For each combination of parameters, we run simulations with all values of $G \in \{10^{-3}, 10^{-2.5}, 10^{-2}, \ldots, 10^{0}\}$. The plot shows the results of all pairs of $G$ (always with the smaller $G$ in the $x$ axis, and the greater $G$ in the $y$ axis). To generate combinations of parameters, we drew random numbers uniformly distributed in the following intervals: $a_{\text{max}} \in [0.1, 5]$, $v_{\text{max}} \in [1, 50]$, $r_{\text{influence}} \in [5, 30]$, $r_{\text{view}} \in [10, 50]$, $t_{\text{prediction}} \in [0, 5]$, $s \in [2, 20]$. For each combination of parameters, we run the simulation both in 2D and 3D. Grey: simulations in 2D. Red: simulations in 3D. See methods for further details about the model.

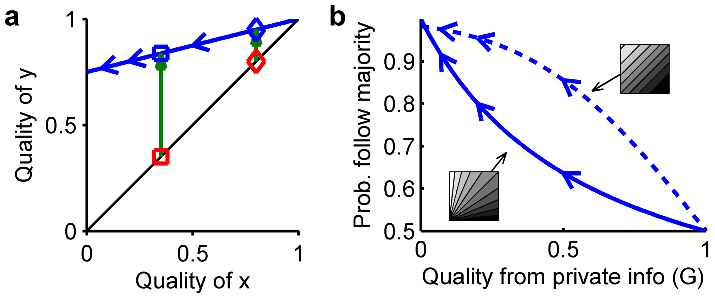


Figure S2: **Selfish herd in the quality landscape. a.** Change in estimated qualities for a selfish herd model (Equation S12). Red: private estimate for favorable conditions (diamond) and adverse conditions (square). Green arrows: contribution of the social information. Blue: trajectory of the final estimated qualities. **b.** For the decision model in (a), probability of choosing the majority option ($y$), as a function of the privately estimated quality ($G$, higher values indicate more favourable conditions). Solid line: relative decision rule. Dashed line: absolute decision rule.

Figure S2a shows the trajectory of this estimation rule for the case of two locations ($M = 2$), when private information modifies the value of $G$. The probability of following the majority increases in adversity both for the relative decision rule (Figure S2b, solid line) and for the absolute one (Figure S2b, dashed line).
